# Noninvasive detection of pancreatic ductal adenocarcinoma in high-risk patients using miRNA from urinary extracellular vesicles

**DOI:** 10.3389/fonc.2025.1682072

**Published:** 2026-01-27

**Authors:** Tomoya Kawase, Yasutaka Kato, Hiroshi Nishihara, Shogo Baba, Tadatoshi Kawasaki, Hiroshi Kurahara, Hideyuki Oi, Shunsuke Kondo, Mao Okada, Tomoyuki Satake, Yukiko Shimoda Igawa, Tatsuya Yoshida, Junji Kita, Johji Imura, Kazuya Kinoshita, Masaya Yokoyama, Atsushi Satomura, Kazuya Takayama, Motoki Mikami, Yumi Nishiyama, Mika Mizunuma, Yuki Ichikawa, Koji Yoshida

**Affiliations:** 1 Department of Gastroenterology and Hepatology, Kawasaki Medical School, Kurashiki, Japan; 2 Department of Gastroenterology, Hokuto Hospital, Obihiro, Japan; 3 Genomics Unit, Keio Cancer Center, Keio University School of Medicine, Tokyo, Japan; 4 Department of Pathology and Genetics, Laboratory of Cancer Medical Science, Hokuto Hospital, Obihiro, Japan; 5 Department of Health Screenings, Hokuto Hospital, Obihiro, Japan; 6 Department of Digestive Surgery, Graduate School of Medical and Dental Sciences, Kagoshima University, Kagoshima, Japan; 7 Department of Experimental Therapeutics, National Cancer Center Hospital, Tokyo, Japan; 8 Department of Hepatobiliary and Pancreatic Oncology, National Cancer Center Hospital, Tokyo, Japan; 9 Department of Medical Oncology, Tokyo Women’s Medical University, Tokyo, Japan; 10 Department of Hepatobiliary and Pancreatic Oncology, National Cancer Center Hospital East, Kashiwa, Japan; 11 Department of Thoracic Oncology, National Cancer Center Hospital, Tokyo, Japan; 12 Department of Surgery, Kumagaya General Hospital, Kumagaya, Saitama, Japan; 13 Department of Diagnostic Pathology, Kumagaya General Hospital, Kumagaya, Saitama, Japan; 14 Department of Surgery, Division of Transplant Surgery, Virginia Commonwealth University, Richmond, VA, United States; 15 Craif Inc., Nagoya, Japan; 16 Institute of Innovation for Future Society, Nagoya University, Aichi, Japan

**Keywords:** liquid biopsy, cancer screening, urinary biomarkers, machine learning, pancreatic ductal adenocarcinoma

## Abstract

Pancreatic cancer (PaC), which is characterized by a high mortality rate, is often diagnosed at an advanced stage, significantly limiting treatment effectiveness. Early detection is crucial for improving survival rates, especially for individuals at high risk (HR) for PaC. Traditional diagnostic methods, including ultrasound, computed tomography, and magnetic resonance imaging (MRI), have limited sensitivity, especially for detecting early-stage PaC. We explored the potential of miRNA from urinary extracellular vesicles (EVs) as a noninvasive diagnostic marker for PaC. An exploratory case–control study was conducted across multiple Japanese institutions. The study included 248 samples from patients with pancreatic ductal adenocarcinoma (PDAC), the most common type of PaC, and HR patients. Differential expression analysis revealed significant differences in 16 miRNAs between the PDAC and HR samples. A machine learning-based algorithm was developed based on these miRNAs to distinguish between PDAC and HR. The algorithm exhibited an AUC of 0.89, a sensitivity of 0.80, and a specificity of 0.79. The algorithm detected the early-stage PDAC (stage 0-IIA) with a sensitivity of 0.73. These findings highlight the potential of the urinary miRNA algorithm as a noninvasive tool to aid in the detection of PDAC, including early-stage cases, in high-risk populations.

## 1 Introduction

Pancreatic cancer (PaC) has a high mortality rate. Despite advances in treatment, the 5-year survival rate of PaC remains approximately 10% in the USA in 2023 (1). Most pancreatic neoplasms (90%) are pancreatic ductal adenocarcinoma (PDAC). Other subtypes include acinar carcinoma, pancreatoblastoma, and neuroendocrine tumors (2). PaC is frequently diagnosed at an advanced stage because early stages are typically asymptomatic. By the time clinical symptoms appear, the malignancy is often advanced and has metastasized to other organs (3). Delayed detection significantly hampers effective treatment and contributes to the high mortality rate. The 3-year survival rate of patients with unresectable invasive PaC is approximately 3% according to a study in Japan (4). In the USA, the 5-year survival rates at stages III and IV PaC are 12% and 3%, respectively (5). Thus, early detection of PaC is essential for improving therapeutic outcomes. Early-stage diagnosis of PaC can significantly improve the 5-year survival rate to over 80% for patients with stage IA PaC (6).

The main risk factors for PaC include type 2 diabetes mellitus (T2DM), chronic pancreatitis, a family history of PaC, intraductal papillary mucinous neoplasm (IPMN), pancreatic cysts, and pancreatic ductal dilation (7). For example, T2DM and chronic pancreatitis were shown to increase the risk of developing PaC by 1.5–2.0 and 16 times, respectively (8, 9). Therefore, screening and monitoring of these high-risk groups are essential for the early detection of PaC. In fact, several medical associations, including Cancer of the Pancreas Screening (CAPS) in 2019 and ASCO in 2018, have established guidelines for the monitoring of HR individuals, emphasizing the crucial role of early-phase detection of PaC (10, 11). A previous study showed that monitoring high-risk individuals increases the likelihood of early detection of PaC and improves 5-year survival rates compared with unmonitored matched controls (12).

Abdominal ultrasound, computed tomography (CT), magnetic resonance imaging (MRI), and endoscopic ultrasonography (EUS) are currently the preferred modalities for PaC screening. Abdominal ultrasound has reported sensitivities of 48–89% but limited specificity and accuracy, with sensitivity decreasing to 30% for small tumors (13). Its diagnostic performance also depends strongly on operator skill (14). Enhanced CT and MRI show overall sensitivities of 89% for detecting PaC (15), but the sensitivity drops markedly to 9.7% for stage 0 disease (16). EUS provides better sensitivity than CT or MRI for early-stage PaC (45.5% for stage 0 and 81.8% for stage I) (16), yet it is invasive and operator-dependent (17). Therefore, developing a noninvasive and accurate screening method for early PaC detection remains an important unmet need.

Liquid biopsy has garnered increasing attention in recent years. Liquid biopsy often provides minimally-invasive or non-invasive tests that are independent of the operator’s skills and can offer cost advantages over tissue biopsy. One of the most widely known liquid biopsy markers for cancer detection is carbohydrate antigen 19-9 (CA 19-9). However, CA 19–9 in the absence of more invasive diagnostic modalities is not a sufficient biomarker of malignancy. Studies have demonstrated that CA 19–9 has a low positive predictive value when used as a screening method for asymptomatic cancer patients (18). To improve the detection of PaC, liquid biopsies that leverage newly discovered biomarkers, such as circulating tumor DNA and miRNA, are in development (19, 20). miRNA, a small noncoding RNA averaging 22 nucleotides in length, plays a crucial role in post-transcriptional gene regulation. miRNA, especially when encapsulated in extracellular vesicles (EVs), have garnered significant attention due to its stability in biofluid and its involvement in cancer pathogenesis (21, 22). EVs secreted by cancer cells are associated with metastasis, and distinct miRNA expression patterns specific to cancer types have been identified in blood (23). Exosomes secreted by cells are partially excreted in the urine during systemic circulation, suggesting that urinary miRNAs could also serve as biomarkers for various cancers (24–26). Because urine can be collected non-invasively, repeatedly, and easily, it could be an ideal sampling fluid for monitoring and screening PaC from HR groups.

Most emerging PaC screening models have been developed by comparing patients with the general population, where the prevalence of cancer is extremely low. Although such approaches, including our previous urinary EV miRNA–based algorithm (77.8% sensitivity and 93.9% specificity) (27), are clinically meaningful, their applicability to large-scale screening is limited due to the high number of false positives and modest efficiency. Focusing on HR individuals offers a more efficient and economically feasible strategy; however, few studies have applied liquid biopsy for HR monitoring.

In this study, we conducted a case–control study across multiple institutions in Japan to explore the feasibility of differentiating PaC from HR based on the machine learning algorithm of miRNAs in urinary EV. We also measured miRNA profiles from the general population without PaC or the risk factors for PaC to investigate the profile similarity between PaC and HR. To the best of our knowledge, this is the first study to compare the miRNA profiles between PaC and HR. We identified several differentially expressed miRNAs between PaC and HR and developed an algorithm to differentiate PaC from HR with a sensitivity of 0.80 and a specificity of 0.79. This algorithm detects early-stage PaC (stage 0-IIA) with a sensitivity of 0.73.

## 2 Materials and methods

### 2.1 Study design and cohorts

This study was approved by the Institutional Review Boards of Craif inc., Hokuto Hospital, Kagoshima University Hospital, Kawasaki Medical School Hospital, Kumagaya General Hospital, and National Cancer Center Hospital. From September 2019 to July 2023, PaC participants from 5 sites; Hokuto Hospital, Kagoshima University Hospital, Kawasaki Medical School Hospital, Kumagaya General Hospital, and National Cancer Center Hospital) and HR participants from 3 sites; Hokuto Hospital, Kagoshima University Hospital, and Kawasaki Medical School Hospital) were recruited. Participants from the general population without PaC or the risk factors for PaC were recruited from 2 sites; Hokuto Hospital and Kumagaya General Hospital. These facilities geographically cover Japan comprehensively. Participants were enrolled so that the age and sex assigned at birth were balanced between PaC and HR, and the stages were approximately evenly distributed. Among the PaC, pathologically confirmed PDAC and high-grade pancreatic intraepithelial neoplasia (PanIN) were classified into a PDAC cohort and the other histological types of PaC were classified into an other-histology cohort. The staging of PaC was performed based on the TNM classification of malignant tumors, 8^th^ edition. HR was defined as patients with any of the following: ductal dilation, T2DM, chronic pancreatitis, pancreatic cysts, IPMN, and family history of PaC. Although pancreatic ductal dilation is often considered a radiologic finding rather than a validated risk factor in Western guidelines (28), we classified it as HR in accordance with the latest Japanese Clinical Practice Guidelines for Pancreatic Cancer, which recognize main pancreatic duct dilation as epidemiologically associated with increased PaC risk (29). In this study, clearly identified serous cystadenomas and other non-neoplastic cystic lesions were excluded, whereas cystic lesions in which IPMN could not be ruled out were included as HR. Participants from the general population were recruited regardless of age and sex. All participants provided written informed consent. The study was conducted in accordance with the Declaration of Helsinki. In the PaC group, urine specimens were collected prior to the initial medical interventions for PaC. All collected urine samples were stored at -80°C on the day of collection, shipped to a facility to measure miRNA, and stored at -80°C until the measurements.

### 2.2 Isolation of extracellular vesicles from urine and RNA extraction

Urine samples (~3.5 mL) were centrifuged at 2,000 g for 30 min at 4°C to remove cells and debris. Subsequently, 3 mL of the resulting supernatant was combined with an equal volume of Total Exosome Isolation Reagent (from urine) (Thermo Fisher Scientific, Waltham, MA) and incubated at room temperature with shaking (100 rpm) for 1 h. The mixture was then centrifuged at 3,000 g for 1 h at 4°C to pellet the EVs. After discarding the supernatant, the pellet was resuspended in 250 μL of phosphate-buffered saline and used for RNA extraction by the MagMAX mirVana Total RNA Isolation Kit (Thermo Fisher Scientific) on a KingFisher Apex System (Thermo Fisher Scientific), following the manufacturer’s protocols. RNA was initially eluted in 30 μL of elution buffer. Given the low RNA concentration, the solution was concentrated using a centrifugal concentrator (Eppendorf, Hamburg, Germany) at 60°C for 30–45 minutes and finally eluted in 5 μL of nuclease-free water (Thermo Fisher Scientific). The RNA extracts were stored at −80°C until further analysis.

### 2.3 Small RNA library construction and sequencing

Libraries were generated from 5 μL of RNA using the QIAseq miRNA Library Kit (QIAGEN, Helden, Germany) according to the manufacturer’s protocol. The concentrations of the prepared libraries were determined using the Qubit™ dsDNA HS Assay Kit (Thermo Fisher Scientific) using a Qubit Flex Fluorometer (Thermo Fisher Scientific), and the libraries were subsequently stored at −20°C for future use. Sequencing was performed on a NovaSeq 6000 System (Illumina, San Diego, CA) with paired-end reads of 150 nucleotides following the guidelines provided by the manufacturer.

### 2.4 Processing of the small RNA sequencing reads

Since miRNAs are typically only 18–25 nucleotides in length, each read in a paired-end setup covers the entire miRNA sequence independently. Therefore, only read one from each pair was used in the analyses. Raw sequencing reads were subjected to initial processing via UMI-tools (30). This involved the extraction of the initial 5′-end bases, indicative of miRNA, which were positioned anterior to the 3’ adaptor sequence. Concurrently, 12 bp unique molecular identifiers (UMIs) succeeding the 3′-adaptor sequence were integrated into the read identifiers to facilitate subsequent analyses. Following this step, reads exceeding 19 bp in length were aligned to the human miRNA reference provided by miRge 3.0 (31) using Bowtie v1.2.3 (32) allowing no mismatch within a 25 bp seed region and no reverse complement mapping. Samples that had more than a total of 10^4^ miRNA total counts were included for the downstream analysis.

### 2.5 Nanoparticle tracking analysis

The size distribution and concentration of EVs were measured using a NanoSight LM10 system (Malvern Panalytical, Worcestershire, UK) by tracking the Brownian motion of individual particles. Six urine samples from the general population were randomly selected for measurement.

### 2.6 Differential expression analysis

DESeq2 (33) was used to identify the differentially expressed miRNAs. The *P* values were adjusted for multiple comparisons by the Benjamini-Hochberg method. The threshold of the adjusted P values was set at *p* = 0.1. No fold-change threshold was applied. miRNAs with at least 2 counts detected for more than 50% of the samples were included in the differential expression analysis. For the pathway enrichment analysis, miRWalk (34) was used. To assess the trend between miRNA expression levels and cancer progression, Spearman’s rank correlation test was conducted between count per million (CPM) normalized miRNA counts and cancer stages (HR, Stage I, Stage II, Stage III, and Stage IV). Stage 0 was removed from this statistical test because of the limited number of cases.

### 2.7 Machine learning

The Dataset was split into 80% of the training set and 20% of the hold-out set so that the age and sex balances were maintained. Several random seeds were tested, and the split that best preserved the balance of cancer stages in PDAC and background diseases in HR was adopted. Prior to the development of the machine learning algorithm, several preprocessing steps were performed in the training set. First, miRNAs with at least 2 counts detected for more than 80% of the samples were incorporated. The percentage threshold of miRNA observed was increased from the differential expression analysis to incorporate more robustly identified miRNAs. The counts of miRNA were normalized by CPM. Feature selection was performed using a bootstrap-based method. The feature candidates included miRNA expression levels, sex, and age. Initially, 30% of the features were randomly selected. The light gradient boosting machine (LightGBM) was then used to conduct 10-fold cross-validation (CV). This process was repeated 50,000 times to generate an average feature importance ranking. From the top of this ranking, features were incrementally added one by one. The feature set that yielded the highest AUC during the 10-fold CV was selected as the optimal set. To develop a prediction model using selected features, a LightGBM was applied. The model was developed to minimize the logarithmic loss. The hyperparameters were adjusted to minimize the logarithmic loss by Optuna (35). The AUC scores, sensitivities, and specificity in the training set were calculated by 10-fold CV. The whole data of the training set was used for the training of the model to apply the model to the hold-out set. To evaluate the algorithm performance across different stages of PDAC, we categorized stages 0 to IIA as an early-stage characterized by localized and resectable tumors, and stages IIB to IV as a late stage defined as unresectable tumors. The same procedure was also applied to other machine learning algorithms such as logistic regression; the results shown are those of the best-performing LightGBM model.

### 2.8 CA19–9 measurement

Plasma CA19–9 levels were measured from 209 patients (134 PDAC and 75 HR) using a chemiluminescent immunoassay at the respective medical facilities when patients were diagnosed. Based on the established clinical threshold for pancreatic cancer detection, samples with CA19–9 levels exceeding 37.0 U/mL were classified as positive.

## 3 Results

### 3.1 Participant demographics and study design

In this study, the PDAC and other-histology cohorts were separated because of their distinct biological and clinical characteristics (36). A total of 255 participants of PDAC and other histology or HR (140 PDAC, 5 other histology, and 110 HR) were enrolled from five institutions in Japan by matching the age and sex distribution. Two samples (1 PDAC and 1 HR) did not satisfy the read count threshold (10^4^ total miRNA counts) and were removed from the analysis set. The remaining 253 samples were used for the final analysis (**Table 1**). In the PDAC cohort, stages I-IV were approximately evenly distributed. Only two participants with stage 0 pancreatic cancer were recruited because of the low prevalence of early-stage diagnosis; one had high-grade PanIN and the other had invasive intraductal papillary mucinous carcinoma (IPMC). In HR, the majority of samples came from patients with the following risk factors: T2DM, IPMN, and family history of PaC. The average age and sex balance of PaC was comparable to the average and balance in the global population of PaC (7). We compared the miRNA profiles of the PDAC and HR and developed a binary classifier. We then applied the comparison of expression profiles and the algorithm to the other-histology cohort (n = 5) in an exploratory manner to assess the applicability of the results to other histological types of PaC. The general population cohort was used as a preliminary reference to evaluate profile similarity between PDAC and HR. Because the general population mostly included healthy participants, the average age was considerably younger than those in the PDAC and HR, and there was a slight sex imbalance (**Table 1**).

**Table 1:** Participant demographics.

Characteristics	PDAC N = 137	other histology N = 4	HR N = 109	General population N = 26
Age^1^	69.8 (10.1)	70.8 (9.8)	70.5 (8.4)	38.7 (18.9)
Unknown				1
Sex				
Male	79 (57%)	3 (60%)	60 (55%)	12 (48%)
Female	60 (43%)	2 (40%)	49 (45%)	13 (52%)
Unknown				1
BMI^1^	22.1 (3.5)	21.4 (3.4)	23.7 (4.4)	23.4 (3.8)
Unknown			3	1
Smoking				
Current use	18 (13%)	1 (20%)	13 (12%)	7 (28%)
Past used	50 (36%)	1 (20%)	42 (40%)	0 (0%)
Never used	70 (51%)	3 (60%)	51 (48%)	18 (72%)
Unknown	1		3	1
Alcohol	60 (43%)	1 (20%)	41 (39%)	14 (56%)
Unknown			5	1
Stage				
0	1 (0.7%)	1 (20%)		
IA	16 (12%)	1 (20%)		
IB	15 (11%)			
IIA	9 (6.5%)	1 (20%)		
IIB	25 (18%)	1 (20%)		
III	31 (22%)			
IV	42 (30%)	1 (20%)		
Histological type				
Ductal adenocarcinoma	138 (99%)			
High grade PanIN	1 (0.7%)			
Adenosquamous carcinoma		1 (20%)		
Carcinoma, undifferentiated		1 (20%)		
IPMC		3 (60%)		
Ductal dilation			15 (14%)	
T2DM			48 (44%)	
Chronic pancreatitis			26 (24%)	
Pancreatic cysts			19 (17%)	
IPMN			51 (47%)	
Family history PaC			43 (39%)	

^1^Mean (SD); n (%).

### 3.2 miRNA profiles among PDAC, HR, and the general population

**Figure 1:**
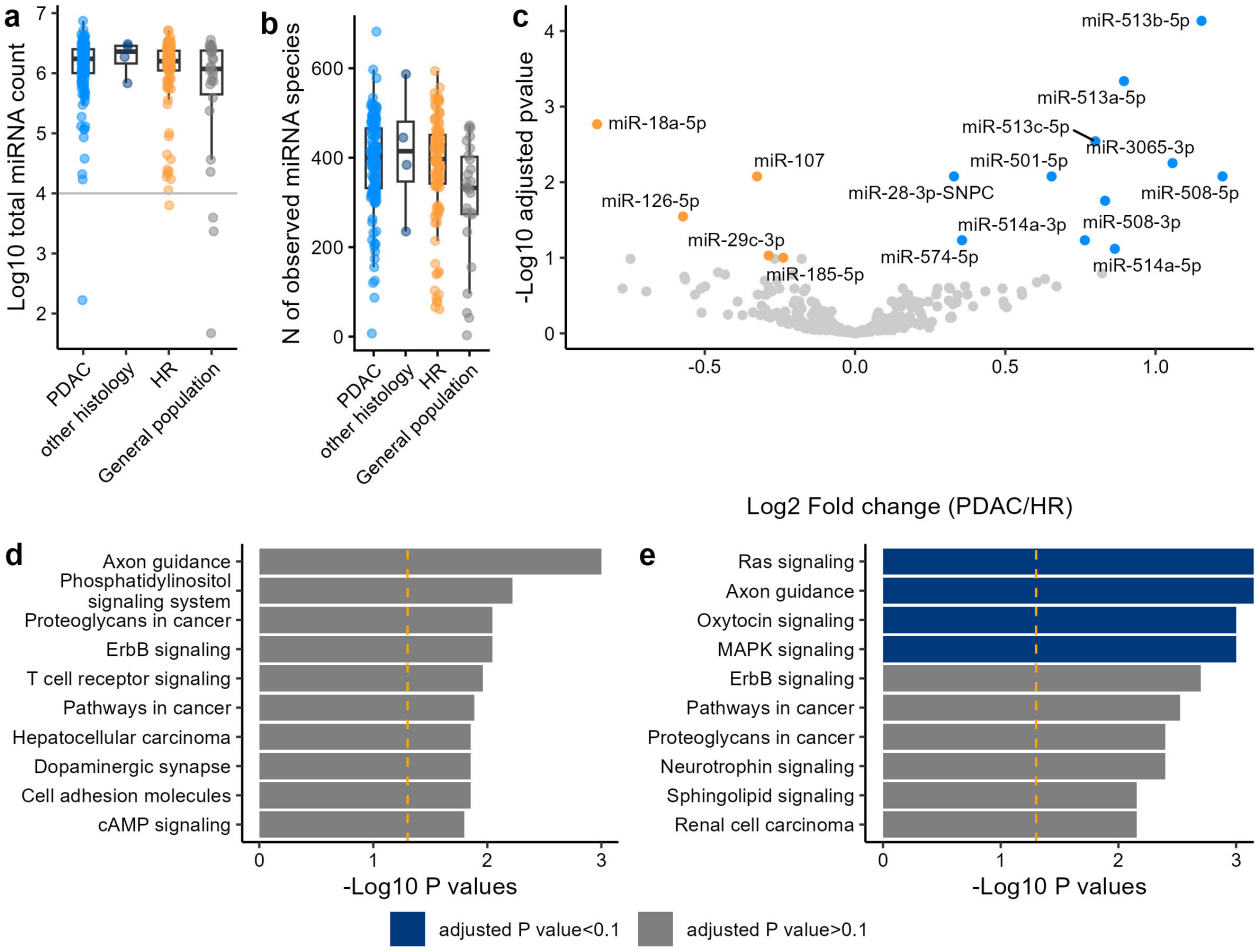
Comparison of miRNA profiles between the PDAC and HR. **(a)** Total counts of UMI deduplicated reads of miRNA in the PDAC, other histology, HR, and general population. Samples with more than 10^4^ miRNA counts (the gray horizontal line) were included for the downstream analysis. **(b)** The number of miRNA species detected in the PDAC, other histology, HR, and general population. **(c)** Differential expression analysis between the PDAC and HR. miRNA with adjusted *P* value <0.1 are highlighted. **(d, e)** Top 10 representative KEGG pathways in significantly upregulated **(d)** and downregulated **(e)** miRNAs.

As an exploratory approach, we compared the miRNA profiles among the PDAC, HR, and general population cohorts to investigate the profile similarity between PDAC and HR. In our EV purification protocol, urinary EV particles with a median peak diameter of 153 nm were obtained, ensuring the enrichment of small extracellular vesicles suitable for miRNA profiling (**Supplementary Figure S1**). The median total miRNA counts (sum of individual miRNA counts per sample) in the PDAC, HR, and general population were 1.73 × 10^6^ and 1.59 × 10^6^, 1.18 × 10^6^, respectively (**Figure 1A**). The median numbers of the observed miRNA species (distinct miRNAs detected with ≥2 counts) were 400, 397, and 333 in the PDAC, HR, and general population, respectively (**Figure 1B**). Differential expression analysis between PDAC and the general population cohorts demonstrated 37 upregulated and 2 downregulated miRNAs in PDAC (**Supplementary Figure S2A**). In HR, we identified 23 upregulated and 3 downregulated miRNAs compared with the general population (**Supplementary Figure S2B**). Intriguingly, among the upregulated miRNAs in PDAC and HR, 20 miRNAs were common (**Supplementary Figure S2C**). Moreover, the log2 fold changes of PDAC and HR relative to the general population showed a strong positive correlation (Pearson ρ = 0.84; **Supplementary Figure S2D**). These results suggest that the miRNA profiles between PDAC and HR are considerably closer to each other than those of the general population, making the differentiation more challenging than between PDAC and healthy controls.

### 3.3 Differential expression analysis

A differential expression analysis between PDAC and HR identified 11 upregulated and 5 downregulated miRNAs in PDAC (**Figure 1C**). Interestingly, many miRNAs such as the miR-513 family (miR-513b-5p, miR-513a-5p, and miR-513c-5p) were upregulated as the cancer stage progressed (**Supplementary Figure S3A**). Likewise, miR-18a-5p, miR-107, and miR-185-5p were downregulated depending on the cancer stage (**Supplementary Figure S3B**). Among the genes targeted by the upregulated miRNAs in the PDAC, none of the associated pathways were statistically enriched (**Figure 1D**). Conversely, 4 growth signal-related pathways were significantly enriched for the genes targeted by the downregulated miRNAs in the PDAC (**Figure 1E**), indicating that the inhibition of these pathways was suppressed in the PDAC. In the other-histology cohort (n = 5), no statistically significant differences were observed compared to the general population (**Supplementary Figures S3C, D**). When PDAC was compared with IPMN, a common and direct precursor lesion to PaC, 18 upregulated and 3 downregulated miRNAs were identified in PDAC (**Supplementary Figure S4A**). Most of the miRNAs identified in the PDAC vs. IPMN comparison were also observed in this analysis, with 8 of 18 upregulated and 2 of 3 downregulated miRNAs overlapping. This is likely because the differentially expressed miRNAs did not show statistically significant variance across different types of risk factors (**Supplementary Figures S4B, C**).

### 3.4 Development of a classifier to detect PDAC from HR

**Figure 2:**
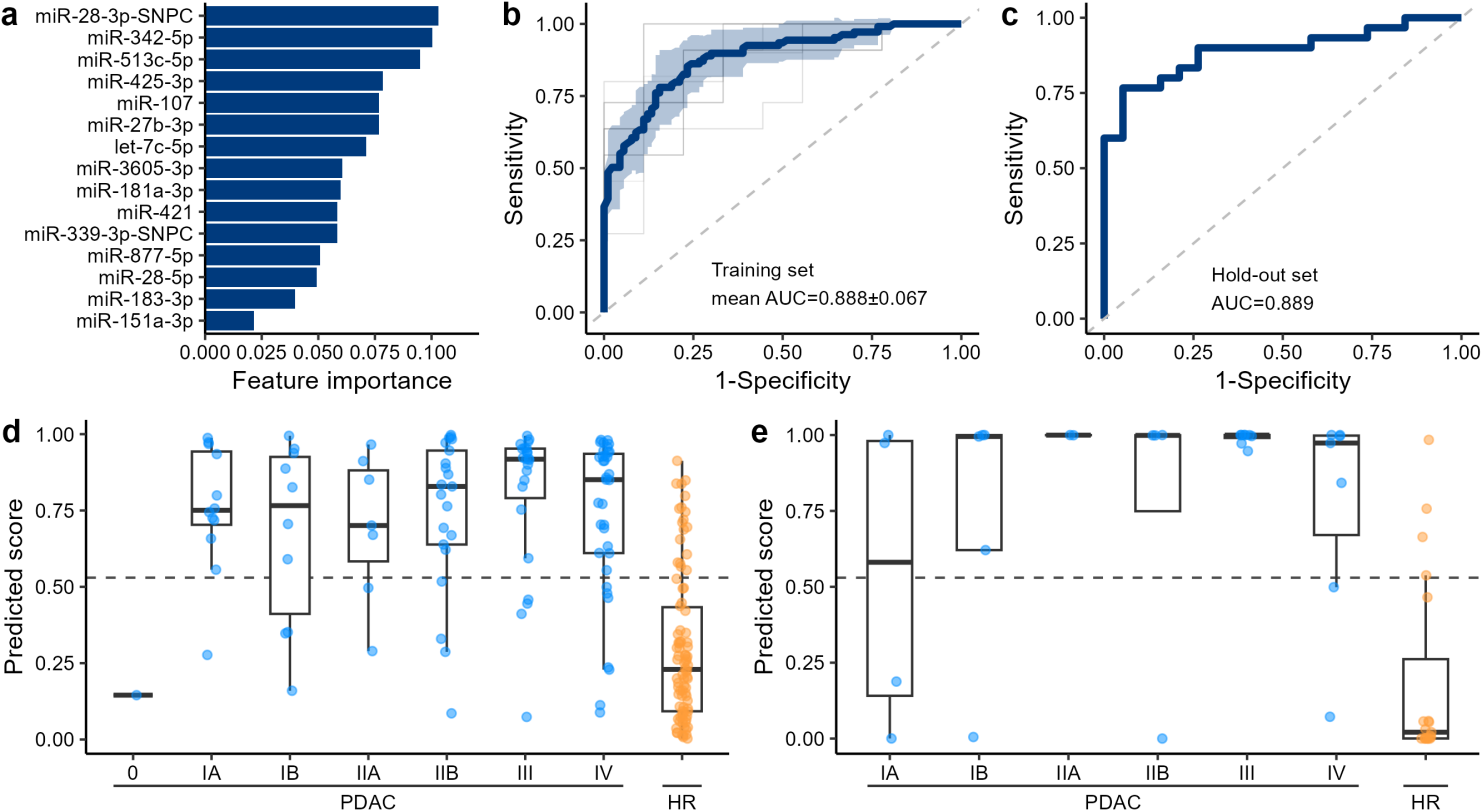
Performance of the LightGBM classifier. **(a)** Feature importance of the LightGBM classifier. The total gains of the splits are shown as feature importance. **(b)** ROC curve of the training set. The mean of 10-fold cross-validation is shown as the solid blue line with SD shown as blue shade. ROC curves in each fold are shown as the thin lines. **(c)** ROC curve of the hold-out set. **(d, e)** Prediction scores of PaC at each stage and HR calculated in the training set **(d)** and hold-out set **(e)**. The dashed lines indicate the threshold to achieve a sensitivity of 0.8 for the training set.

**Figure 3:**
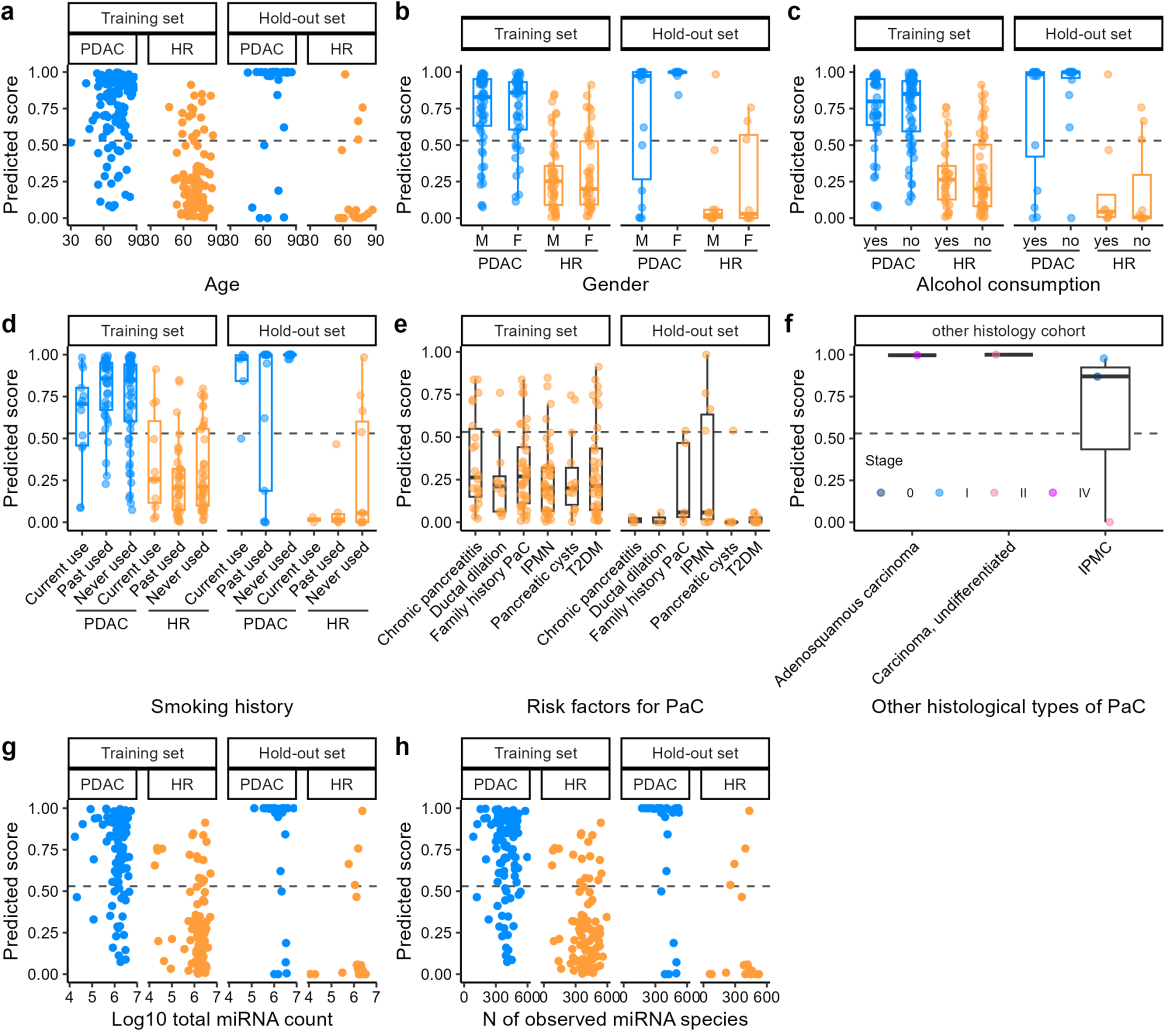
Prediction scores of the PDAC and HR. **(a-h)** Prediction scores of PaC and HR over **(a)** age, **(b)** sex (M: male, F: female), **(c)** alcohol consumption, **(d)** smoking history, **(e)** risk factors in HR, **(f)** other histology, **(g)** total counts of UMI deduplicated reads of miRNA in PDAC and HR, and **(h)** the number of miRNA species detected in PDAC and HR. The dashed lines indicate the threshold to achieve a sensitivity of 0.8 for the training set. Yasutaka Kato is an advisor and has stock options of Craif Inc. Mika Mizunuma and Yuki Ichikawa are board members and shareholders of Craif Inc. Atsushi Satomura, Kazuya Takayama, Motoki Mikami, and Yumi Nishiyama are employees and have stock options of Craif Inc. Other authors have no conflicts of interest.

To develop a machine learning-based algorithm for the detection of PDAC from HR, the cohort was split into a training and hold-out set (**Supplementary Table S1**). We started from a systematic feature selection by bootstrapping (see Materials & Methods). A LightGBM binary classifier was developed from the 15 miRNA features after feature selection (**Figure 2A**). The performance of the algorithm was evaluated via a 10-fold CV, and the AUC of the ROC curve was 0.888 (SD = 0.067) (**Figure 2B**). The AUC in the remaining hold-out set was 0.889 (**Figure 2C**). A slight decrease in the prediction scores for early-stage PDAC (stage 0-IIA) compared with late-stage PDAC (stage IIB-IV) was observed in the training set (**Figure 2D**) and hold-out set (**Figure 2E**). The cancer prediction scores were not largely biased by age, sex, alcohol consumption habits, smoking history, risk factors of HR, miRNA total count, and the number of observed miRNA species (**Figures 3A–E, G, H**). When the threshold of the prediction scores was set at 0.530 to adjust the sensitivity in the training set to 0.8, the sensitivity and specificity in the training set were 0.798 (SD = 0.120, 95% CI: 0.712–0.884) and 0.800 (SD = 0.126, 95% CI: 0.710–0.890), respectively (**Table 2**). At this cutoff, the overall sensitivity and specificity in the hold-out set were 0.800 (95% CI: 0.627–0.905) and 0.789 (95% CI: 0.567–0.915), respectively. For early-stage and late-stage PDAC, the sensitivity scores were 0.750 (SD = 0.326, 95% CI: 0.517–0.983) and 0.813 (SD = 0.128, 95% CI: 0.721–0.905) in the training set, and 0.727 and 0.842 in the hold-out set, respectively. When applied in an exploratory manner to the other-histology cohort (n = 5), the algorithm predicted 4 out of 5 cases to be above the cutoff of 0.530 (**Figure 3F**), with a sensitivity of 0.80 (4/5). As previously reported (18), CA 19–9 measured in this study also demonstrated that early-stage PDAC showed lower CA19–9 levels than late-stage PDAC (**Supplementary Figure S5A**). The overall sensitivity of CA19–9 was 0.634 and the specificity was 0.747 (**Supplementary Table S2**). For the early stage, the sensitivity decreased to 0.341, whereas the sensitivity of the late stage was 0.808. The prediction score from the algorithm developed from miRNA and CA19–9 showed a week correction with a Pearson correlation coefficient of 0.275, suggesting that the miRNA in the urinary EVs reflects independent systematic conditions from CA19-9 (**Supplementary Figure S5B**).

**Table 2:** Detection scores of PDAC.

Metric	Training set	Hold-out set
Sensitivity	0.798 ± 0.120 (95% CI 0.712–0.884)	0.800 (95% CI: 0.627–0.905)
Sensitivity (Early stage)	0.750 ± 0.326 (95% CI 0.517–0.983)	0.727 (95% CI 0.393 –0.936)
Sensitivity (Late stage)	0.813 ± 0.128 (95% CI 0.721–0.905)	0.842 (95% CI 0.604 –0.966)
Specificity	0.800 ± 0.126 (95% CI 0.710–0.890)	0.789 (95% CI: 0.567–0.915)

## 4 Discussion

This is the first exploratory study that utilizes urinary biomarkers to discriminate between PDAC and HR. We identified miRNAs from urinary EVs that were significantly upregulated and downregulated in the PDAC compared with the HR (**Figure 1C**, **Supplementary Figure S3**). The algorithm developed in this study discriminated PDAC from HR with an AUC of 0.889 in the hold-out set. The sensitivity for detecting early-stage PDAC was 0.750 in the training set and 0.727 in the hold-out set, which are higher than that of CA19–9 in this study (**Supplementary Table S2**) and comparable or higher than the sensitivity reported in previous reports (13).

Urine has the potential to serve as an alternative body fluid with comparable diagnostic performances to blood, as shown in this study along with a previous study on urinary cell-free DNA (37). Blood samples can be influenced by hemolysis (38). Additionally, blood collection is often painful and typically needs to be performed in a clinical setting, which is more burdensome for patients and can result in poor compliance. In contrast, urine collection is painless, noninvasive, and can be done at home in most cases, facilitating routine testing. This makes urine a more convenient option for monitoring people at risk for PaC. However, urine sampling conditions can vary with hydration and disease status, which may affect the miRNA profiles. Some urine samples in this study showed low miRNA yields (**Figure 1A**), due to variations in urine conditions such as dehydration. In our previous study, we proposed a standardized collection method involving the use of first morning urine or the avoidance of excessive fluid intake before collection (39). Applying this approach in future studies is expected to further improve model performance and data consistency.

In the current diagnostic protocol for PaC, clinical symptoms such as abdominal pain and loss of appetite, blood test results, and imaging findings, together with known risk factors, are considered to determine the need for further testing. When PaC is suspected, CT, MRI, or EUS are performed. However, because early-stage PaC is often asymptomatic and blood tests such as CA19–9 lack sufficient sensitivity (**Supplementary Figure S5A**), patients are not routinely referred for diagnostic procedures that could detect treatable cancers. Abdominal ultrasound, CT, MRI, and EUS are the main imaging modalities currently used for PaC detection, but all have limitations in identifying early-stage disease. Abdominal ultrasound has reported sensitivities of 48–89% and specificity of 40–91%, with sensitivity decreasing to 30% for small tumors (<2 cm) (13, 14). Enhanced CT and MRI show overall sensitivities of 89% for detecting PaC (15), but the sensitivity drops to 9.7% for stage 0 disease (16). EUS offers better detection performance, with sensitivities of 45.5% for stage 0 and 81.8% for stage I (16), but it remains invasive and highly operator-dependent (17). These limitations highlight the need for an accurate and noninvasive method capable of detecting early-stage PaC, especially in high-risk (HR) individuals. In this study, the classifier’s sensitivity for all PDAC stages and early-stage PaC were 0.800 and 0.727, respectively. These results suggest potential utility, although direct comparisons with existing imaging modalities should be made cautiously given differences in methodology and cohort characteristics. The Japanese Ministry of Health, Labour and Welfare (MHLW) set the positive predictive value (PPV) targets for cancer screening tests in 2023 at 2.5%, 3.0%, and 4.1% for gastric, colorectal, and lung cancers, respectively. Assuming a 1% prevalence of PaC in HR individuals, a test with 80% sensitivity and 80% specificity yields an estimated PPV of 3.9% and NPV of 99.7%, indicating that these parameters are acceptable and comparable to those of established cancer screening programs. The intended clinical use of this urinary miRNA-based test is comparable to current tumor markers and imaging surveillance for people at higher risk of PaC, serving as a bridge between initial screening and diagnostic procedures.

EVs in the bloodstream undergo filtration in the renal glomeruli, are reabsorbed in the renal tubules, reach the bladder, and are released in the urine. Cancer tissue, especially in the early stages, is very small and may not significantly impact the miRNA profile. How then does cancer tissue change urinary miRNA profiles? A previous study using a breast cancer cell line demonstrated that tumor cells released approximately 10 times more exosomes than normal cells (40). Another study observed greater enrichment of miRNA in cancer exosomes than miRNA from normal cells (41). However, the hypothesis that only small tumor cells at the early stages significantly influence the miRNA profiles in urine through the release of large amounts of EVs and miRNA might be less plausible, given the current understanding of EVs and miRNA. We hypothesize that blood and urinary miRNA profiles are also influenced by responses from surrounding stromal and immune cells, in addition to tumor cells. For instance, in PaC, bone marrow mesenchymal stem cells have been reported to secrete EVs that contain a certain miRNA to inhibit cancer proliferation (42, 43). In other studies, natural killer cells were reported to secrete EVs that contained miRNA that inhibited cancer progression (44). These findings support the hypothesis that both blood and urinary miRNA profiles are influenced by the systematic conditions and not only by cancer tissues. We hypothesized that this characteristic of urinary miRNA explains the similarity in miRNA expression profiles between PDAC and other histology (**Figure 2SD**), as well as the similarity observed between the overall HR cohort and IPMN (**Supplementary Figure S4**), and supports the applicability of the PDAC-based algorithm to the other-histology cohort (**Figure 3F**).

We identified five downregulated miRNAs in PDAC (**Figure 1C**). Previous studies have demonstrated that miR-126, one of the downregulated miRNAs in PaC in this study, is related to inflammation and is inhibited in cancer (45). Another study showed that the downregulation of miR-185-5p causes cancer proliferation (46). Unlike the downregulated miRNAs, the association between PaC and the upregulated miRNAs has not been extensively reported. Interestingly, miR-513-5p, one of the miRNAs induced depending on the stage of PDAC in our study (**Supplementary Figure S3A**), has been shown to have reduced expression in many different cancers such as gastric cancer, lung cancer, and colon cancer (47–49). In PaC, various types of PaC cell lines have been demonstrated to have lower levels of miR-513b-5p and its suppression promoted the invasion and migration (50). The miR-513-5p family may have originated from immune cells that responded to tumorigenesis and progression. Reports indicate that several miRNAs are downregulated in cancer tissue but upregulated in the blood of cancer patients. For example, the miR141/200c was shown to be downregulated in PaC tissues due to the promoter hypermethylation (51), while these miRNAs were upregulated in the plasma of patients with PaC (52).

To further explore the biological significance of the identified miRNAs, we summarized their reported functions and associated pathways in **Supplementary Table S3**. The majority of the upregulated miRNAs are also dysregulated in other malignancies, participating in canonical oncogenic pathways such as Wnt/β-catenin, PI3K/AKT, or TGF-β signaling. In contrast, several downregulated miRNAs act as tumor-suppressive regulators involved in angiogenesis, cell-cycle control, and MAPK signaling, whose loss may facilitate PDAC progression. Collectively, these results imply that the urinary miRNA profile integrates both tumor-specific molecular alterations and systemic host responses, providing complementary biological insights into tumor–host interactions.

This exploratory study has several limitations. First, the number of cases required for algorithm development may need careful consideration due to the relatively small differences in miRNA expression levels between the PDAC and HR (**Figure 1C**). Specifically, the number of stages 0, I, and II participants in this study (N = 1, 31, and 34, respectively) may not be sufficient. Consequently, the training of early-stage cases became unstable, as reflected by a standard deviation of 0.326 in the training data. The algorithm we developed in this study exhibited a slightly lower performance in early-stage PaC (**Figures 2D, E** and **Table 2**). This is likely due to the expression levels of some miRNAs being dependent on the cancer stage (**Supplementary Figure S3**). To increase the performance and robustness for early-stage PDAC, a greater number of cases of the early-stage PaC might be needed. Second, to fairly evaluate the performance and robustness of this algorithm for early- and late-stage PaC, validation studies will be necessary. In this study, comorbidities such as T2DM were not recorded for patients with PaC because clinical documentation prioritized pancreatic cancer–related treatment. Future validation studies should also pay attention to the balance of comorbidities. Third, the general population was much younger than the PaC and HR. This age difference likely contributed to variations in the proportions of smokers and drinkers among the groups. These confounding effects limit the interpretation of the profile similarity between PaC and HR. However, there were no differentially expressed miRNAs between the general population younger than 40 and older or equal to 40, implying that age is not a large cofounding factor for urinary miRNA profiles (**Supplementary Figure S2E**).

## Data Availability

The datasets presented in this article are not readily available because public data sharing was not explicitly addressed in the informed consent obtained from the participants. Requests to access the datasets should be directed to Yuki Ichikawa.
