# Supplementary material for: Noninvasive detection of pancreatic ductal adenocarcinoma in high-risk patients using miRNA from urinary extracellular vesicles
Source: Front Oncol. 2026 Jan 27;15:1682072. doi: 10.3389/fonc.2025.1682072 (PMC12886021; doi:10.3389/fonc.2025.1682072)
Supplement: Supplementary file 1 [file DataSheet1.docx]

Supplementary Material

# Supplementary Figures


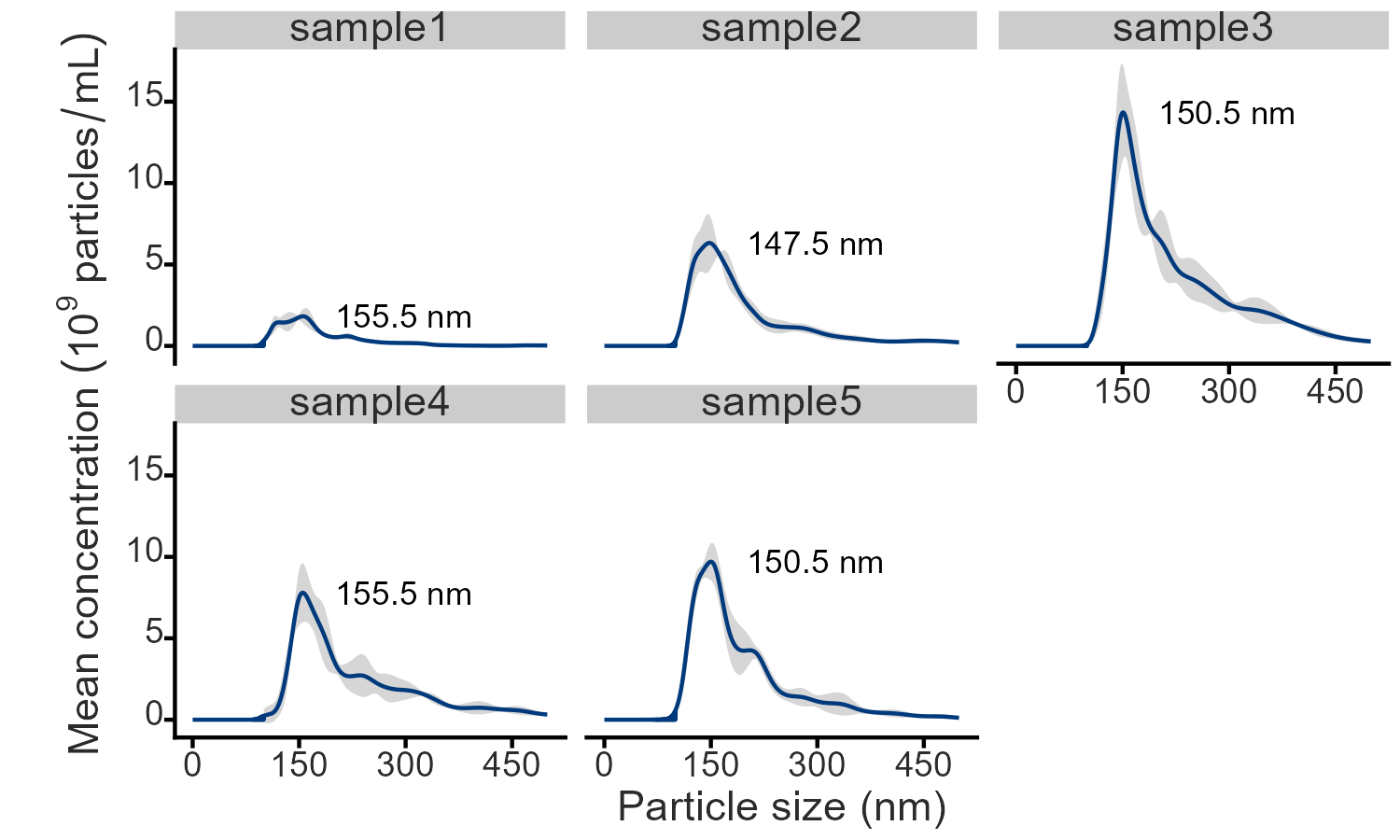


**Supplementary Figure 1.** Particle size distribution of five samples from the general population. The shaded ribbon indicates the ±SD across five replicate NTA measurements per subject. Peak particle sizes, shown in each panel, were determined as the particle size corresponding to the maximum mean concentration for each subject.


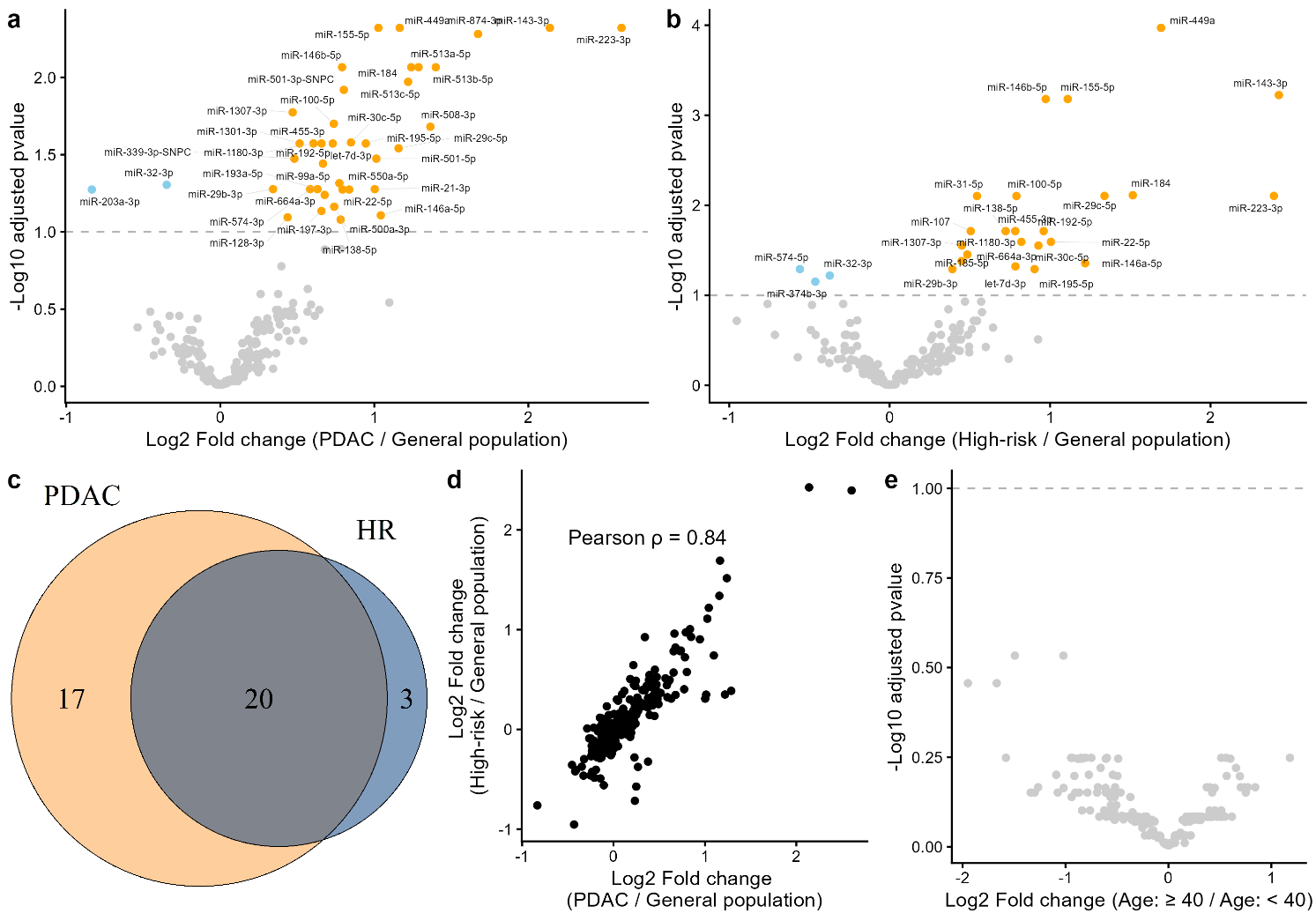


**Supplementary Figure 2.** miRNA expression profiles among the PDAC, HR, and general population. (a, b) Differential expression analysis between the PDAC and general population (a) and the HR and general population (b). (c) The number of shared and unique upregulated miRNAs detected in PDAC (a) and HR (b) compared to the general population. (d) Comparison of log2 fold changes in miRNA expression between HR and the general population, and between PDAC and the general population. The Pearson correlation coefficient between the two sets of fold changes is shown. (e) Differential expression analysis between the general population aged younger than 40 and older or equal to 40.

**
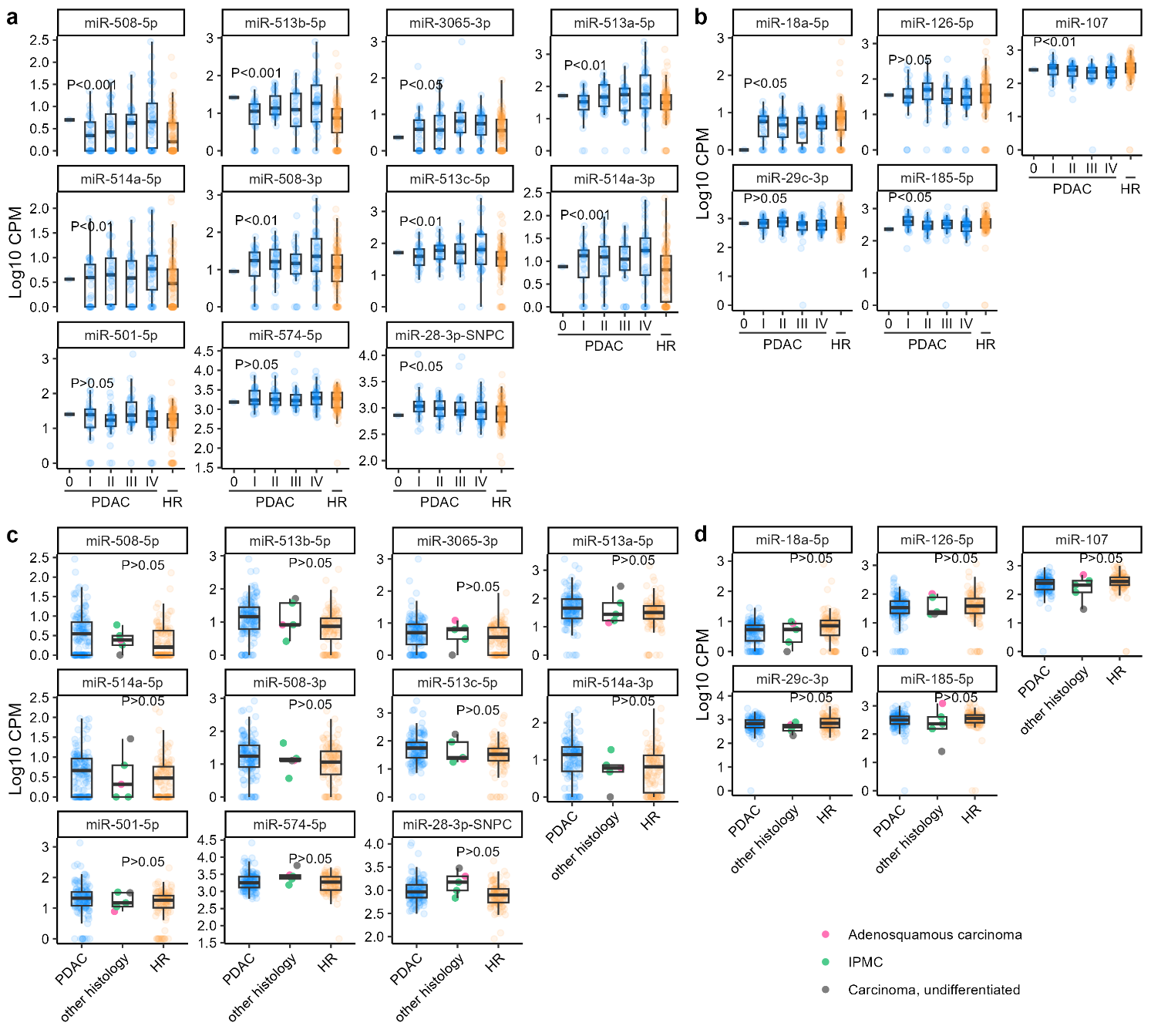
**

**Supplementary Figure 3.** Expression levels of differentially expressed miRNA in the PDAC at each stage and HR. (a, b) Expression levels of significantly upregulated (a) and downregulated miRNA (b). Adjusted *P* values calculated by Spearman's rank correlation test between the miRNA expression levels and cancer stages (HR, Stage I, Stage II, Stage III, and Stage IV) are shown. Stage 0 was removed from the statistical analysis. (c, d) Expression levels of significantly upregulated (c) and downregulated miRNAs (d) over the histological types of PaC and HR. *P* values were calculated using the Wilcoxon rank-sum test between the other histology and HR cohorts, followed by multiple comparison adjustments using the BH method.

**
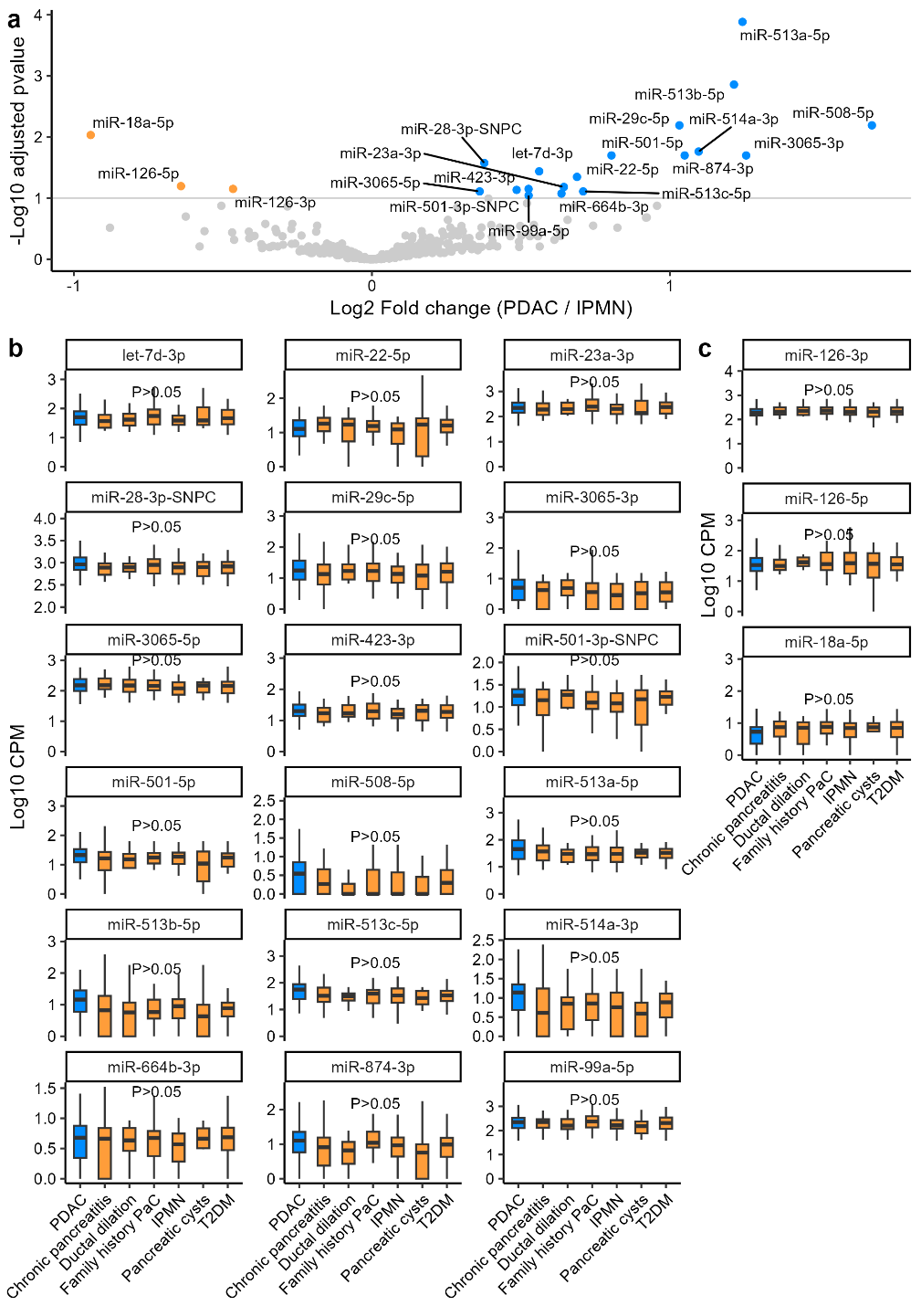
**

**Supplementary Figure 4.** Comparison between PDAC and IPMN. (a) Differential expression analysis between PDAC and IPMN. (b, c) Expression levels of significantly upregulated (b) and downregulated miRNA. *P* values were calculated using the ANOVA test among risk factors in HR, followed by multiple comparison adjustments using the BH method.

**
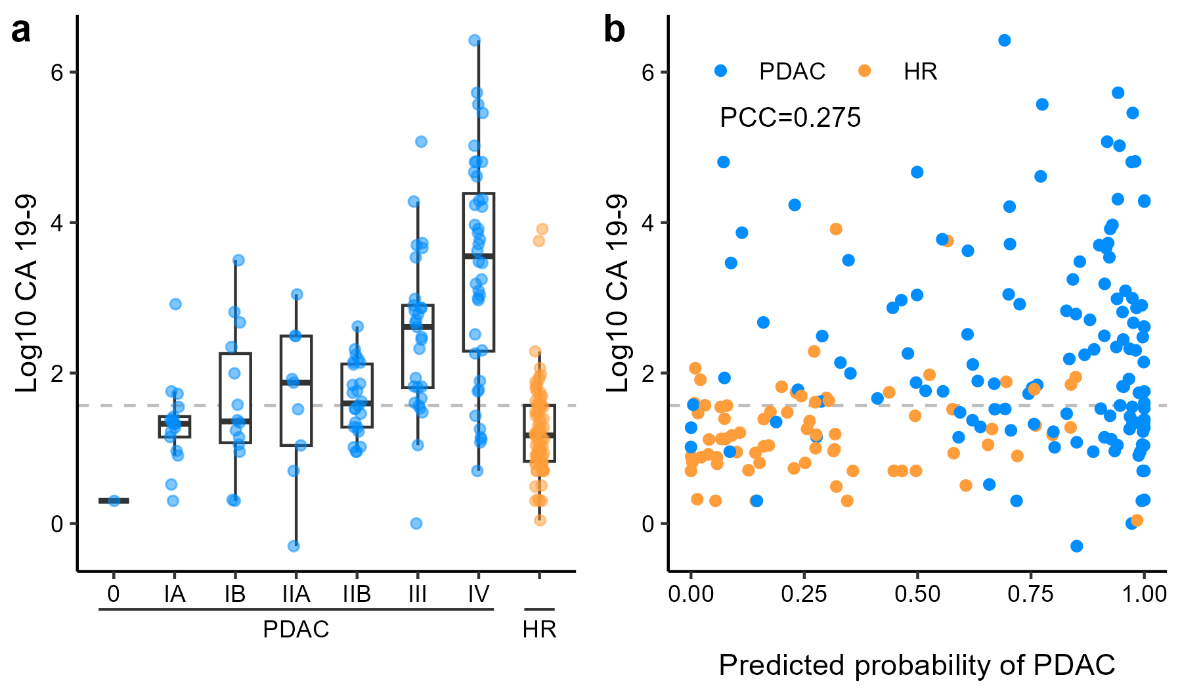
**

**Supplementary Figure 5.** CA 19-9. (a) CA 19-9 values over each stage of PaC and HR. (b) Scatter plot between CA 19-9 levels and prediction scores of PDAC.

| Table S1 Participant demographics of training and hold-out set | | | | |
| --- | --- | --- | --- | --- |
|  | **PaC** | | **HR** | |
|  | **Train**  N = 109^1^ | **Hold-out**  N = 30^1^ | **Training**  N = 90^1^ | **Hold-out**  N = 19^1^ |
| **Age^1^** | 69.9 (10.2) | 69.5 (10.0) | 70.6 (8.3) | 70.1 (9.1) |
| **Sex** |  |  |  |  |
| Male | 61 (56%) | 18 (60%) | 49 (54%) | 11 (58%) |
| Female | 48 (44%) | 12 (40%) | 41 (46%) | 8 (42%) |
| **BMI^1^** | 22.2 (3.6) | 21.9 (3.1) | 23.7 (3.9) | 23.8 (6.2) |
| Unknown |  |  | 2 | 1 |
| **Smoking** |  |  |  |  |
| Current use | 13 (12%) | 5 (17%) | 11 (13%) | 2 (11%) |
| Past used | 37 (34%) | 13 (45%) | 36 (41%) | 6 (32%) |
| Never used | 59 (54%) | 11 (38%) | 40 (46%) | 11 (58%) |
| Unknown | 0 | 1 | 3 | 0 |
| **Alcohol** | 44 (40%) | 16 (53%) | 33 (39%) | 8 (42%) |
| Unknown |  |  | 5 | 0 |
| **Stage** |  |  |  |  |
| 0 | 1 (0.9%) | 0 (0%) |  |  |
| IA | 12 (11%) | 4 (13%) |  |  |
| IB | 10 (9.2%) | 5 (17%) |  |  |
| IIA | 7 (6.4%) | 2 (6.7%) |  |  |
| IIB | 21 (19%) | 4 (13%) |  |  |
| III | 23 (21%) | 8 (27%) |  |  |
| IV | 35 (32%) | 7 (23%) |  |  |
| **Histological type** |  |  |  |  |
| Ductal adenocarcinoma | 108 (99%) | 30 (100%) |  |  |
| High grade PanIN | 1 (0.9%) | 0 (0%) |  |  |
| **Ductal dilation** |  |  | 12 (13%) | 3 (16%) |
| **T2DM** |  |  | 38 (42%) | 10 (53%) |
| **Chronic pancreatitis** |  |  | 22 (24%) | 4 (21%) |
| **Pancreatic cysts** |  |  | 14 (16%) | 5 (26%) |
| **IPMN** |  |  | 41 (46%) | 10 (53%) |
| **Family history PaC** |  |  | 38 (42%) | 5 (26%) |
| ^1^Mean (SD) | | | | |

| Table S2 CA 19-9 scores | |
| --- | --- |
|  | **CA19-9** |
| **Sensitivity** | 0.634 |
| **Sensitivity (Early stage)** | 0.341 |
| **Sensitivity (Late stage)** | 0.808 |
| **Specificity** | 0.747 |

Table S3**:** miRNAs Dysregulated in PDAC

| **miRNA** | **Expression in PDAC** | **Pathway / Target** | **Notes** |
| --- | --- | --- | --- |
| **miR-501-5p** | Up | TGF-β pathway (target: TGFBR3) (1) | Tumor-associated macrophage-derived exosomes; delivered to PDAC cells, promote invasion/metastasis via TGFBR3 suppression and TGF-β signaling activation (1). |
| **miR-513a-5p** | Up | Apoptosis regulation (target: BCL2L1, anti-apoptotic factor) (1) | Sponged by lncRNA MIR4435-2HG; normally suppresses BCL2L1 to promote apoptosis as a tumor-suppressive miRNA. In PDAC, overexpression of MIR4435-2HG inhibits miR-513a-5p, leading to increased BCL2L1 and enhanced tumor cell survival (1). |
| **miR-513b-5p** | Up | PI3K/Akt pathway (target: PIK3R3) (2) | A tumor-suppressive miRNA that, when downregulated, leads to PI3K pathway activation and enhanced invasion/proliferation. In PDAC, it is sponged by the lncRNA FTX, resulting in reduced miR-513b-5p activity and increased proliferation/invasion; FTX knockdown restores miR-513b-5p and promotes apoptosis (2,3) |
| **miR-513c-5p** | Up | PTEN/AKT pathway (target: KLF10, a transcription factor) (4) | Oncogenic miRNA; high expression in PDAC is associated with poor prognosis. FLVCR1-AS1 (lncRNA) sponges miR-513c-5p, relieving suppression of KLF10 (↑PTEN, ↓AKT) and thereby inhibiting tumor growth and metastasis. In contrast, high miR-513c-5p expression suppresses KLF10, activating the AKT pathway and promoting malignancy (4) |
| **miR-514a-3p/5p** | Up | Proliferation/EMT regulation (target: EGFR) (5) | Originally derived from the Xq27.3 cluster and generally tumor-suppressive across various cancers. For example, in renal cell carcinoma, it directly suppresses EGFR to inhibit EMT (5) |
| **miR-508-3p/5p** | Up | EMT and inflammatory signaling pathways (targets: ZEB1, NFKB1, etc.) (5) | Typically functions as a strong tumor suppressor miRNA, inhibiting EMT and stemness via ZEB1 and SALL4, and directly suppressing NF-κB signaling by targeting NFKB1. Although upregulated in PDAC, this contrasts with downregulation in other cancers such as esophageal SCC, suggesting context-dependent functional variation (5). |
| **miR-3065-3p** | Up | Stemness maintenance (target: CRLF1) (6) | Oncogenic miRNA. In colorectal cancer, it promotes cancer stemness and metastatic potential by targeting CRLF1 (a cytokine receptor-like factor) (6) |
| **miR-574-5p** | Up | Cell proliferation / therapy resistance (target: CTDSP1)(7) | Functions as an oncomiR; suppresses CTDSP1 (RNA Pol II phosphatase), activates the Rb/E2F pathway, and promotes cell-cycle progression and proliferation. Elevated during gemcitabine resistance acquisition in PDAC, suggesting involvement in chemoresistance and inflammatory signaling (TLR8 activation) (8). |
| **miR-18a-5p** | Down | KRAS pathway (target: KRAS) (9) | Normally represses KRAS expression; part of the oncogenic miR-17–92 cluster, but reported to be downregulated in PDAC (9) |
| **miR-107** | Down | Cell cycle (target: CDK6) (9) | Tumor-suppressive miRNA; can be epigenetically silenced. Inhibits CDK6 to block G1-phase progression. Downregulated in PDAC tissue and plasma; low expression correlates with advanced disease (T/N stage, liver metastasis) and poor prognosis (10). Restoration suppresses PDAC cell proliferation, indicating therapeutic potential (9). |
| **miR-126-5p** | Down | Invasion / metastasis suppression (target: ADAM9) (11) | A potent tumor-suppressive miRNA. In PDAC cells, miR-126 reduces ADAM9 (a membrane-bound metalloprotease) expression, markedly decreasing migration and invasion (11). |
| **miR-98-5p** | Down | MAPK/ERK pathway (target: MAP4K4) (12) | Tumor-suppressive member of the let-7 family. Strongly downregulated in PDAC; its target MAP4K4 becomes upregulated, activating ERK signaling and promoting tumor growth and metastasis (12). Also directly suppresses VEGFA, inhibiting angiogenesis. miR-98 generally acts as a suppressor, and its loss in PDAC releases proliferative and pro-angiogenic pathways. |
| **miR-28-3p** | Down | Hedgehog / EMT pathway (target: ARF6) (13) | Context-dependent miRNA. In gastric cancer, miR-28-3p targets ARF6 to inhibit the Hedgehog pathway, suppressing EMT and metastasis. Conversely, in lung cancer (14), it promotes proliferation via PTEN inhibition. Reported to be downregulated in PDAC. |

**References**

1. Zhang W, Xing J, Liu T, Zhang J, Dai Z, Zhang H, Wang D, Tang D. Small extracellular vesicles: from mediating cancer cell metastasis to therapeutic value in pancreatic cancer. *Cell Communication and Signaling* (2022) 20:1. doi: 10.1186/s12964-021-00806-y

2. Jin W, Liang Y, Li S, Lin G, Liang H, Zhang Z, Zhang W, Nie R. MiR-513b-5p represses autophagy during the malignant progression of hepatocellular carcinoma by targeting PIK3R3. *Aging* (2021) 13:16072–16087. doi: 10.18632/aging.203135

3. Li S, Zhang Q, Liu W, Zhao C. Silencing of FTX suppresses pancreatic cancer cell proliferation and invasion by upregulating miR-513b-5p. *BMC Cancer* (2021) 21:290. doi: 10.1186/s12885-021-07975-6

4. Lin J, Zhai S, Zou S, Xu Z, Zhang J, Jiang L, Deng X, Chen H, Peng C, Zhang J, et al. Positive feedback between lncRNA FLVCR1-AS1 and KLF10 may inhibit pancreatic cancer progression via the PTEN/AKT pathway. *Journal of Experimental & Clinical Cancer Research* (2021) 40:316. doi: 10.1186/s13046-021-02097-0

5. Yoshida K, Yokoi A, Yamamoto Y, Kajiyama H. ChrXq27.3 miRNA cluster functions in cancer development. *Journal of Experimental & Clinical Cancer Research* (2021) 40:112. doi: 10.1186/s13046-021-01910-0

6. Li Y, Xun J, Wang B, Ma Y, Zhang L, Yang L, Gao R, Guan J, Liu T, Gao H, et al. miR-3065-3p promotes stemness and metastasis by targeting CRLF1 in colorectal cancer. *J Transl Med* (2021) 19:429. doi: 10.1186/s12967-021-03102-y

7. He Z, Ji Y, Yuan Y, Liang T, Liu C, Jiao Y, Chen Y, Yang Y, Han L, Hu Y, et al. Uncovering the role of microRNAs in esophageal cancer: from pathogenesis to clinical applications. *Front Pharmacol* (2025) 16: doi: 10.3389/fphar.2025.1532558

8. Gu J, Zhang J, Huang W, Tao T, Huang Y, Yang L, Yang J, Fan Y, Wang H. Activating miRNA-mRNA network in gemcitabine-resistant pancreatic cancer cell associates with alteration of memory CD4+ T cells. *Annals of Translational Medicine* (2020) 8:279–279. doi: 10.21037/atm.2020.03.53

9. Supadmanaba IGP, Mantini G, Randazzo O, Capula M, Muller IB, Cascioferro S, Diana P, Peters GJ, Giovannetti E. Interrelationship between miRNA and splicing factors in pancreatic ductal adenocarcinoma. *Epigenetics* (2022) 17:381–404. doi: 10.1080/15592294.2021.1916697

10. Imamura T, Komatsu S, Ichikawa D, Okajima W, Ohashi T, Kiuchi J, Nishibeppu K, Ikoma H, Taniguchi H, Otsuji E. Depleted Tumor Suppressor miR-107 in Plasma Relates to Tumor Progression and Is a Novel Therapeutic Target in Pancreatic Cancer. *Journal of the American College of Surgeons* (2017) 225:e120. doi: 10.1016/j.jamcollsurg.2017.07.848

11. Hamada S, Satoh K, Fujibuchi W, Hirota M, Kanno A, Unno J, Masamune A, Kikuta K, Kume K, Shimosegawa T. MiR-126 Acts as a Tumor Suppressor in Pancreatic Cancer Cells via the Regulation of ADAM9. *Mol Cancer Res* (2012) 10:3–10. doi: 10.1158/1541-7786.MCR-11-0272

12. Hazari V, Samali SA, Izadpanahi P, Mollaei H, Sadri F, Rezaei Z. MicroRNA-98: the multifaceted regulator in human cancer progression and therapy. *Cancer Cell International* (2024) 24:209. doi: 10.1186/s12935-024-03386-2

13. Ji H, Liu S, Yang L, Wu Y, Zhang H, Liu X, Li L, Li L. miR-28–3p suppresses gastric cancer growth and EMT-driven metastasis by targeting the ARF6/Hedgehog axis. *Molecular and Cellular Probes* (2025) 79:102010. doi: 10.1016/j.mcp.2025.102010

14. Cui F, Zhou Q, Xiao K, Qian H. MicroRNA‑28 promotes the proliferation of non‑small‑cell lung cancer cells by targeting PTEN. *Mol Med Rep* (2020) 21:2589–2596. doi: 10.3892/mmr.2020.11033
